# Tus, an *E. coli* Protein, Contains Mammalian Nuclear Targeting and Exporting Signals

**DOI:** 10.1371/journal.pone.0008889

**Published:** 2010-01-26

**Authors:** Stanislaw J. Kaczmarczyk, Kalavathy Sitaraman, Thomas Hill, James L. Hartley, Deb K. Chatterjee

**Affiliations:** 1 Protein Expression Laboratory, Advanced Technology Program, SAIC-Frederick, Inc., National Cancer Institute at Frederick, Frederick, Maryland, United States of America; 2 Department of Microbiology and Immunology, School of Medicine and Health Sciences, University of North Dakota, Grand Forks, North Dakota, United States of America; The Scripps Research Institute, United States of America

## Abstract

Shuttling of proteins between nucleus and cytoplasm in mammalian cells is facilitated by the presence of nuclear localization signals (NLS) and nuclear export signals (NES), respectively. However, we have found that Tus, an *E. coli* replication fork arresting protein, contains separate sequences that function efficiently as NLS and NES in mammalian cell lines, as judged by cellular location of GFP-fusion proteins. The NLS was localized to a short stretch of 9 amino acids in the carboxy-terminus of Tus protein. Alterations of any of these basic amino acids almost completely abolished the nuclear targeting. The NES comprises a cluster of leucine/hydrophobic residues located within 21 amino acids at the amino terminus of Tus. Finally, we have shown that purified GFP-Tus fusion protein or GFP-Tus NLS fusion protein, when added to the culture media, was internalized very efficiently into mammalian cells. Thus, Tus is perhaps the first reported bacterial protein to possess both NLS and NES, and has the capability to transduce protein into mammalian cells.

## Introduction

Replication of the circular *E. coli* chromosome starts at the origin of replication (*oriC*), and proceeds bidirectionally to a region on the opposite side called *Ter*, where replication is terminated [1], [2]. Within the *Ter* region are found ten short (∼20 bp) DNA sequences, called “*Ter*” sites, which are similar but not identical (3). Binding of Tus, a 35 kDa trans-acting protein, to the *Ter* sequences blocks movement of the DNA replication complex in an orientation-specific manner: *Ter* sites (with bound Tus) in the permissive orientation allow the replication complex to pass, whereas Tus/*Ter* complexes in the non-permissive orientation block its movement [4].

The binding of Tus to the *TerB* site, with an observed binding constant (K_obs_) of 3–10×10^−13^ M [5], is one of the strongest known DNA–protein interactions involving a monomeric protein. The crystal structure of the Tus-*Ter* complex showed that Tus protein binds duplex DNA tightly through a novel two-domain structure with inter-domain β-strands and cannot be released without a large conformational change of the Tus protein [6]. A total of 42 amino acid residues out of the 309 amino acids of Tus interact with the DNA. Of these, 18 residues lie within the β-strands (FGHI), which lie partially within the major groove of the *Ter* DNA, and the remaining 24 residues are dispersed along the Tus protein between amino acids 50 and 302. Alterations of some of the amino acids in these domains exhibit partial or complete loss of DNA binding or DNA replication arrest activity [7], [8], [9].

In mammalian cells active transport of proteins into the nucleus is facilitated by the presence of a nuclear localization signal (NLS) that is recognized by and associated with nuclear import receptors [10]–[13]. Similarly, nuclear export of proteins is facilitated by the presence of a nuclear export signal (NES), in association with an export receptor, probably CRM-1 [13], [14] in human cells. Although the presence of nuclear targeting sequences are common in mammalian proteins, the widely used Cre recombinase of bacteriophage P1 [15] and VirD2 of Agrobacterium [16] are among only a few prokaryotic proteins known to contain a sequence that functions as an NLS. However, there was no report to show that these prokaryotic proteins contain NES, in addition to NLS in the same protein. We show here with GFP fusion constructs that the *E. coli* Tus protein contains both NLS and NES motifs that function efficiently in human cells. Tus may be the first prokaryotic protein known to carry both nuclear import and nuclear export sequences. However, since Tus is a DNA binding protein, the presence of both NLS in Tus is probably fortuitous; and thus, the notion of biological significance would be purely speculative.

Several eukaryotic proteins have the ability to travel through biological membranes. Examples include the HIV-1 TAT protein, the herpes simplex virus 1 (HSV-1) DNA-binding protein VP22, and the Drosophila Antennapedia (Antp) homeotic transcription factor [17]. The small protein transduction domains (PTDs) from these proteins can be fused to other macromolecules, peptides, or proteins to successfully transport them into a cell [17], [18]. So far, there has been no example of any prokaryotic proteins or peptides derived from prokaryotic proteins to perform a similar function. Here we show that full-length Tus protein or a peptide (NLS) is capable of transporting proteins into mammalian cells from the culture media. The NLS and NES of Tus protein may be important in transducing proteins into mammalian cells.

## Methods

### Plasmid Constructions

All DNA manipulations were performed using standard procedures. The wild-type Tus protein gene was cloned from the *E .coli* chromosome by PCR using the primers, 5′-ATT TTA GCT AGC GGA GGT GCG CGT TAC GAT CTC GTA GAC CGA CTC-3′ (oligo 1) and 5′-TAT ATT CAA TTG TTA ATC TGC AAC ATA CAG GTG CAG CCG TG-3′ (oligo 2) containing NheI and MunI restriction enzyme sites (underlined), respectively. The PCR product was cloned into a derivative of a Gateway destination vector, pDest 47, (Invitrogen) at the NheI-MunI sites and designated pDest 472-Tus. GFP was cloned into this vector by site-specific recombination using Gateway (Invitrogen) cloning technology. The final expression vector containing GFP-Tus fusion was under a strong CMV promoter. The entire fusion construct was verified by sequencing. The plasmid is called pDest GFP-Tus.

Amino terminal deletion derivatives of Tus protein were cloned by replacing the full-length Tus from pDest 472-GFP-Tus with PCR fragments. To clone amino acids 217 to 309 and 264 to 309 of Tus at the end of GFP, oligos 5′-ATA TTT GCT AGC GAT ATC GCT GCC CTG CCA CAG AAC-3′ and 5′-ATA TT T GCT AGC ATT AAT CGG GAT AAT GGC GC-3′, respectively were used with oligo 2 above as the 3′ oligo for PCR. The restriction site NheI is underlined. For carboxy terminal end deletions, oligo 1 (above) was used in conjunction with the following oligos: 5′ATA ATA CAA TTG TTA TAA ATG GCG GAA ATG ACG CAA CGC 3′ (amino acids 1–77), 5′ATA ATA CAA TTG TTA GAG TTC TGA TTC AAC CGT GAC G 3′ (amino acids 1–133), 5′ATA ATA CAA TTG TTA TGA TTT CAG GCT TTT TTC CAG CTG TG 3′ (amino acids 1–195). The PCR fragments were cloned at NheI and MunI sites of pDest 472-GFP-Tus to replace the full-length Tus. All clones were sequence-verified. To clone amino acids 1–217 of Tus, the plasmid pDest 472-GFP-Tus was cut with EcoRV and MunI, filled in with Klenow fragment and the largest fragment was self-ligated.

To clone the NLS of Tus, we designed the following oligos: 5′- CTAGCAAGTTAAAAATCAAACGTCCGGTGAAGTAATAAC-3′ and 5′-AATTGTTATTACTTCACCGGACGTTTGATTTTTAACTTG-3′. The underlined bases are complementary to NheI and MunI restriction site following digestion. The oligos were annealed using standard procedure and ligated to pDest GFP-Tus digested with NheI and MunI. This replaced the full-length Tus with the NLS sequence.

We have also generated SV40 NLS (SPKKKRKVE) fused to GFP to compare with GFP-Tus NLS for protein translocation into the nucleus. To clone SV40-NLS oligos (upper oligo 5′-CTAGCCCGAAGAAAAAACGTAAAGTGGAGTAATAAC-3′ and lower oligo 5′-AATTGTTATTACTCCACTTTACGTTTTTTCTTCGGG-3′) were annealed and ligated to pDest GFP-Tus similar to Tus NLS as above. The plasmids sequences were verified and purified; and PC3 cells (ATCC) were transfected by standard procedure (see below).

### Mutation of Tus Proteins

Mutation of Tus was done using the “QuickChange” procedure developed by Stratagene (California). To change the amino acid K277 to A227 (K227A), the oligos used were 5′-GCTGCCCTG CCACAGAACGCAGCGCTAAAAATCAAACGTCCGGTG3′ (top oligo) and 5′-CACCGGACGTTTGATTTTTAGCGCTGCGTTCTGTGGCAGGGCA- GC-3′ (bottom oligo); a restriction site, AfeI (underlined), was created to screen the mutants. For the K229A mutation, the oligos were 5′- GCCCTGCCACAGAACGCGAA GCTAGCAATCAAACGTCCG-GTGAAG-3′ (top oligo) and 5′CTTCACCGGACGTTT- GATTGCTAGCTTCGCGTTCTGTGGCAGGGC-3′ (bottom oligo), a restriction site, NheI (underlined), was created to screen mutants. For K231A, the oligos used were 5′- CAGAACGCGAAGTTAAAAATCGCGCGCCCGGTGAAGGTGCAGCCG-3′ (top oligo) and 5′- CGGCTGCACCTTCACCGGGCGCGCGATTTTTAACTTCG- CGTTCTG-3′ (bottom oligo); a restriction enzyme BssHII site (underlined) was created for screening mutants. For R232A mutation the following oligos were used, 5′- CAG AAC GCGAAGTTAAAAATCAAGGCGCCGGTGAAGGTGCAGCCG-3′(top) and 5′- CGGCTGCACCTTCACCGGCGCCTTGATTTTTAACTTCGCGTTCTG-3′, a restriction site, KasI (underlined), was created for easy screening. All mutations and clones were verified by DNA sequencing.

### Cell Culture, Transfection and Microscopy

A human prostate carcinoma cell line PC3 (ATCC) was cultured in DMEM (Invitrogen) supplemented with 10% FCS and antibiotics. Cells were plated 24 hours before transfection at 10^6^ cell per well (in six-well culture dishes) in final volume of 2 ml of complete culture medium. The following day, 2 hours before transfection, culture medium was replaced with 2 ml of fresh, complete culture medium. Plasmids coding for GFP-Tus fusion proteins were diluted at 2 µg/10 µl in TE buffer and used for transfection using the FuGene-6 transfection reagent (Roche Biochemicals).

After transfection (24 hours), gene expression was monitored using a set of fluorescence filters specific for GFP detection with ZEISS fluorescence microscopy. Images were archived using a SPOT-2 image camera.

### Purification of Histidine-Tagged Fusion Protein

*E. coli* cells containing his-tagged fusion protein were in rich medium with ampicillin at 100 µg/ml at 30°C. Induction of expression was done using 1 mM IPTG for 4 hours. Cells were harvested by centrifugation at 10,000 g at 4°C. Cells were washed with (equal volume of cell culture) Ni-NTA binding/lysis buffer (50 mM NaPO_4_ pH 8.0, 300 mM NaCl, 10 mM immidazole and 20% glycerol). Cells were collected by centrifugation as before.

We resuspended 5 gm of cells in Ni-NTA binding/lysis buffer (4 ml/gm of cells) containing 2 mg/ml lysozyme and incubated in an ice bath for 45 minutes, inverting tube every 30 seconds. Cells were then transferred to 37°C water bath for 5 minutes. Cells were sonicated 6X with 10 seconds burst, with 10 seconds rest. RNAseA was added to 10 µg/ml and DNase was added to 5 µg/ml to the extract. The extract was incubated at 37°C for 5 minutes, inverting tube every 30 seconds. The extract was transferred to the ice bath for 5 minutes. The extract was centrifuged at 10,000 g for 30 minutes at 4°C. The supernatant (Fraction I) was collected.

An Ni-NTA column (5 ml) was set up in a cold room and equilibrated with 5 column volumes of binding buffer. Fraction I sample was loaded onto column at a flow rate of approximately 1 drop/20 seconds (0.3 ml/minute). Flow-through was collected in a tube.

The column was washed with 10 column of wash buffer (binding buffer containing 20 mM immidazole). Unbound/washed protein was collected in a beaker. Bound protein was eluted with elution buffer (binding buffer containing 250 mM immidazole) by collecting 0.3 ml fractions. Each fraction was checked for protein concentration by a Bio-Rad protein assay, followed by polyacrylamide gel analysis. Fractions containing half-maximal peak with the lowest contamination of hi-tagged fusion proteins were pooled (Fraction II), dialyzed against 500 ml dialysis buffer (50 mM NaPO_4_, pH 8.0, 150 mM NaCl, and 20% glycerol). Protein concentration of the dialyzed Fraction II was determined. 50 µl aliquots of Fraction II were prepared and stored at −80°C. We obtained greater than 95% pure Tus fusion protein using this method.

### Delivery of Fusion Protein into PC3 Cells

The PC3 cells were plated at 1×10^5^ per well in 6-well in tissue culture plates 24 hours before transduction. The next day, the culture medium was replaced with 2 ml of fresh medium containing 7.5 µg Tus-GFP, Tus-GFP-NLS, Tus-Cre, or TAT-Cre protein to each well. For control experiments, we treated the cells with the same amount of GFP (without NLS). At indicated time points, cells were washed in PBS, and the green fluorescence was observed under fluorescence microscopy.

### DAPI Staining

Plates containing adherent cells were washed with PBS, and fresh PBS supplemented with DAPI at 1∶1000 dilution of stock solution (1 mg/mL) was added to plates; plates were incubated for10 minutes in tissue culture incubator. After this time, plates were washed again with PBS, and fresh, complete culture medium was added. The blue fluorescence was detected under fluorescence microscopy using a DAPI filter.

## Results and Discussion

### Nuclear Import and Export of GFP-Tus Fusion

**Figure 1:**
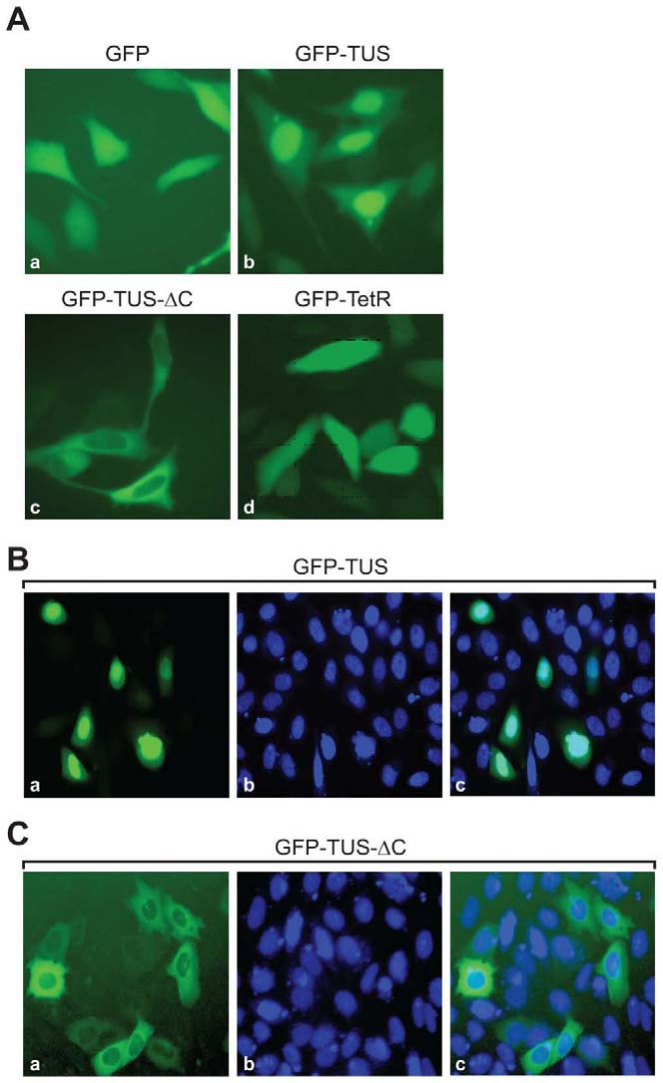
Subcellular localization of GFP and GFP-Tus derivatives. A Panel (a), GFP only; Panel (b), GFP-TUS is the fusion of GFP with full-length Tus; Panel (c), GFP-TUS-ΔC is GFP-fused with amino acids Tus containing 1–217 of Tus; (d), GFP-TetR is the fusion of GFP with a non specific protein, tetR. B. Panel (a), GFP fluorescence of GFP-Tus protein; Panel (b), cells were stained with DAPI; and Panel (c), Superimposition of panels (a) and (b). C. Cytoplasmic localization of GFP fused with 1–217 amino acids of Tus. Panel (a), GFP fluorescence; Panel (b), DAPI staining; and Panel (c), Superimposition of panels (a) and (b).

The Tus protein was fused to the carboxy terminus of GFP, as described in Materials and Methods. The fusion was made initially as a model to develop a novel protein microarray-on-demand technology [19]. For this purpose, we wanted to examine the expression of the GFP fusion in mammalian cells by transient expression. GFP expression (pDest 472-GFP) in PC3 cells alone showed fluorescence distributed throughout the cells (Figure 1A[a]). In contrast, transfection of PC3 cells with pDest 472-GFP-Tus resulted in strong green fluorescence almost exclusively in the nucleus of the cells (Figure 1A[b]). On the other hand, GFP fusion of the carboxy-end deleted version of Tus showed localization of the fusion protein to cytoplasm of PC3 cells (Figure 1[c]). To conclusively determine nuclear localization of GFP-Tus protein, following transfection, cells were examined for green fluorescence, as well as for DAPI nuclear staining. The results as shown in Figure 1B and 1C clearly suggest that GFP-Tus fusion protein is indeed localized in the nucleus. Since Tus is a bacterial protein, specific targeting to the nucleus was unexpected. Nuclear pores in eukaryotic cells consist of 9–10 nm-diameter channels that might allow smaller proteins (<40 kDa), to diffuse in and out of the nucleus freely, but import of larger proteins is an active process [12]. The size of the GFP-Tus fusion protein is about 62 kDa and thus, it would be difficult for this large fusion protein to pass the nuclear membrane by simple diffusion. In addition, to show that nuclear or cytoplasmic localization is due to the presence of Tus or of a carboxy-end deleted version of Tus, respectively, we cloned GFP with another protein, tetR, which has no known NLS on NES with GFP. As can be seen in Figure 1(d), the GFP fluorescence is present all over the cells. The results suggest that specific localization of the GFP fusion protein is due to the presence of the NLS and NES sequence in Tus.

### Location of Nuclear Localization Signal in Tus

**Figure 2:**
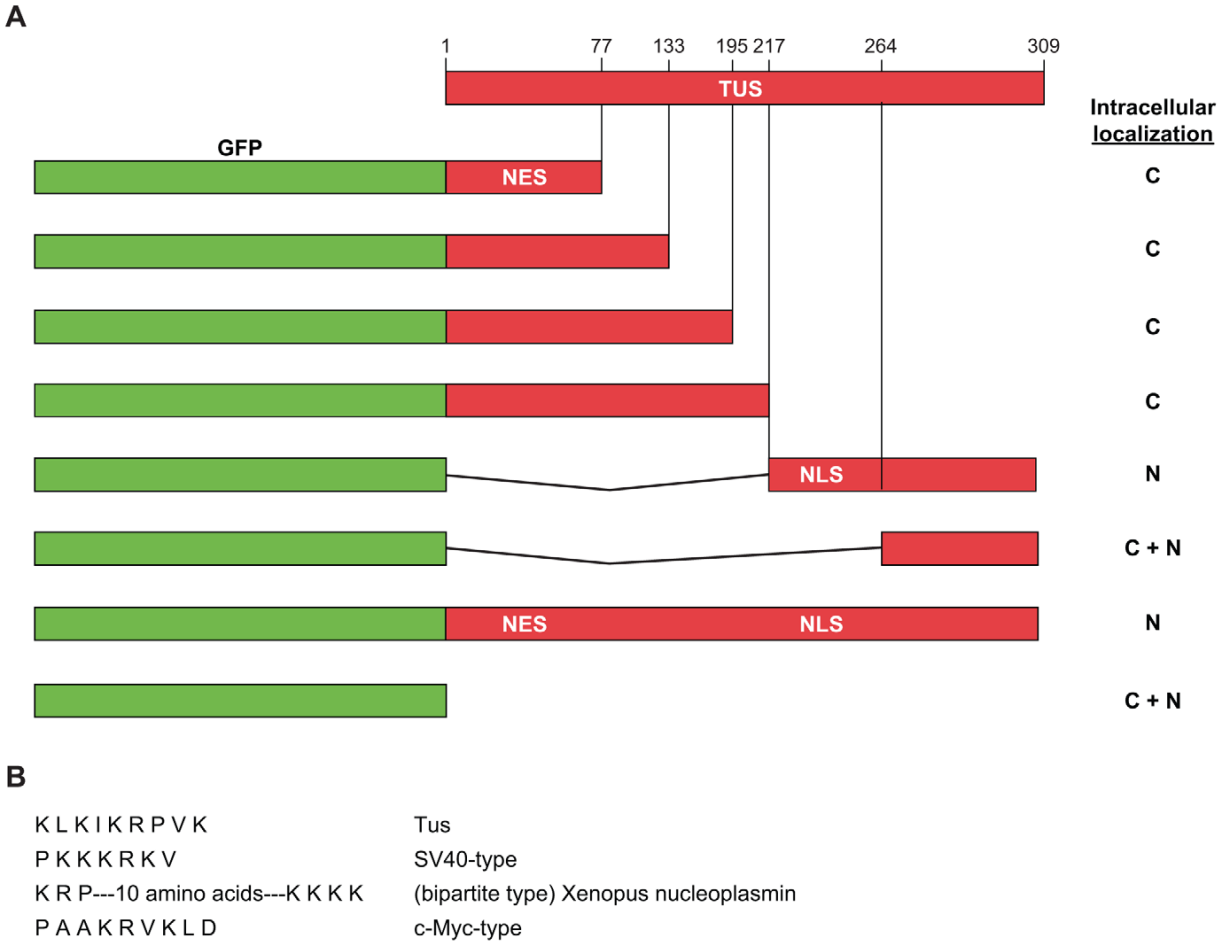
A. Localization of NLS and NES determinants of Tus by deletion analysis. A. Full-length and various deletions of Tus were fused with GFP, and localization of the fusion protein in nucleus (N), cytoplasm (C), and all over the cell (C+N) were indicated on right. The boundary of the NLS region was between amino acids 218 and 264 and the boundary of the NES region was between 1 and 77 amino acids. B. Comparison of various types of known NLS with putative NLS sequence of Tus.

To identify the location of the nuclear localization signal (NLS) within Tus, we constructed a series of N- and C-terminal deletion mutants of Tus, which were fused to GFP to determine the subcellular distribution of green fluorescence in the PC3 cell line. Transfection of PC3 cell lines with all of these fusion constructs was performed, and the results are summarized in Figure 2A.

The results suggest that fusion of up to 217 amino acids from the N-terminus of Tus protein (full-length 309 amino acids) with GFP causes loss of nuclear targeting. However, the fusion of amino acids 218 to 309 of Tus to the C-terminus of GFP restored nuclear targeting. Thus, the nuclear targeting signal must be located within the 92 amino acids at the C-terminus of Tus. Fusion of the Tus fragment corresponding to amino acids 264 to 309 with GFP did not result in nuclear targeting. Therefore, the location of the NLS must be confined within 47 amino acids (218–264) near the carboxy end of Tus.

Nuclear localization signals do not fit a tight consensus but generally fall into two classes: short stretches of four to seven basic (SV40 type) amino acids [18]; and longer bipartite sequences comprising two stretches of basic amino acids separated by several less conserved spacer amino acids [20]. We did not detect any bipartite-like nuclear localization sequences within amino acids 218 to 264. However, after scanning the amino acid sequence, we detected a short stretch of basic amino acids resembling an NLS-like sequence of KLKIKRPVK (amino acids 227–235) as shown in Figure 2B. We speculated that this sequence might be the putative nuclear targeting signal in Tus. In the crystal structure of Tus, this region is present in the β-strand H, one of the interdomains of Tus protein that forms the main elements of *Ter* DNA sequence recognition.

### Mapping of Nuclear Localization Signal (NLS)

**Figure 3:**
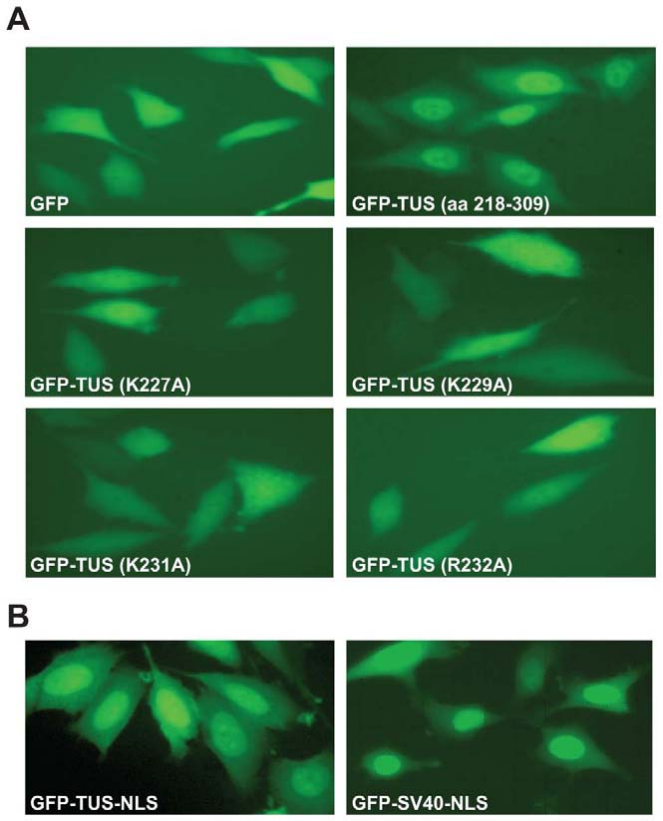
Mutational analysis of NLS region of Tus. Mutation was done using a plasmid containing GFP fused to Tus (218–309) expressed in PC3 cells. Expression of only GFP was shown in the top left panel. Localization of un-mutated GFP-Tus (218–309) fusion was shown in the upper right panel. Mutants (indicated) of specific amino acids Tus within 218–309 amino acids fused to GFP were expressed in PC3 cells. **B.** Tus-NLS region containing 9 amino acids and SV40-NLS was fused to GFP expressed in PC3 cells (positive control).

To further demonstrate the importance of the basic amino acids in the putative NLS-like sequence, we systematically mutated them and examined the subcellular distribution of green fluorescence following transfection into PC3 cells. The construct used for the mutation contains amino acids 218–309 of Tus fused to GFP. As can be seen in Figure 3A, alteration of any one of the basic amino acids resulted in pronounced perturbation of nuclear targeting of the fusion proteins. These results suggest that each of these basic amino acids plays a crucial role in nuclear transport. Finally, we cloned amino acids KLKIKRPVK (amino acids 227 to 235), at the end of GFP and showed that these amino acids are all that is needed for nuclear transport of GFP (Figure 3B). For comparison with a positive control, we also cloned a SV40 NLS, a highly characterized NLS motif, similar to Tus NLS with GFP. As can be seen, GFP fluorescence is very similar in both cases (Figure 3B), suggesting that both NLS sequences function similarly.

It has been proposed that NLS sequences often overlap with the nucleic acid binding domain of proteins [21]. It is true that the residues KLKIKRPVK of Tus protein are indeed involved in interacting with *ter* sequences [8]. However, our tests show it is very unlikely that the DNA binding activity of Tus is essential for nuclear targeting. Purified GFP fusion protein containing Tus residues 218–309 showed no apparent binding affinity towards *Ter* (our unpublished observation), yet it still localized to the cell nucleus. The loss of *Ter* binding activity was expected, since important amino acids in the interdomain β-strands FG [8] and at other locations in the Tus protein were missing in the deleted construct. Thus, our results suggest that for nuclear targeting, the NLS sequence must have basic amino acids but need not bind nucleic acids.

A bacteriophage DNA binding protein, Cre, and VirD2 of Agrobacterium were shown to possess NLS sequences [16], [22]. However, unlike Tus, Cre has a bipartite-like NLS sequence and VirD2 has both mono- and a bipartite-like NLS sequence. There are some other subtle differences with respect to the location of NLS in these two proteins. In VirD2, the NLS is present at the carboxy terminus, while in the Cre, the bipartite-like NLS is present at the amino terminus. In VirD2, two regions encompassing 417–420 and 430–434 were needed for NLS function. However, Cre needs two long regions, Region I and Region II, encompassing about 172 (100–271) amino acids for nuclear targeting. Interestingly, Tus only needs a short stretch of basic amino acids for nuclear targeting, very similar to most mammalian-type NLS sequences. Active nuclear import of proteins is believed to occur in two steps: binding to cytoplasmic surface of the nuclear pore complex, followed by the energy-dependent translocation into the nucleus [9], [14], [23]. Experiments are underway to understand the mechanism of nuclear transport in eukaryotic cells mediated by Tus.

### Identification of Nuclear Export Signal (NES)

**Figure 4:**
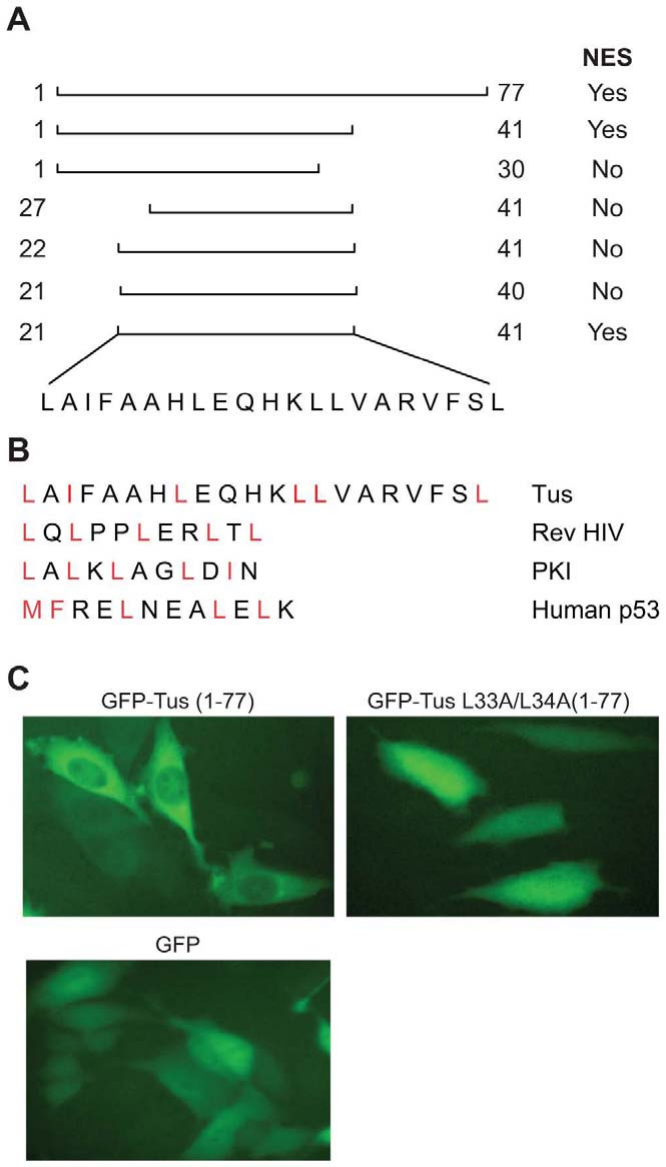
A. Location of NES sequence, comparison of various NES and mutational analysis of NES region of Tus. A. Replacement of full-length Tus in pDest 472 GFP-Tus vector with the indicated region of Tus was used to localize the region of NES. B. Comparison of Tus NES with known NES (p53, HIV Rev and PKI) were shown. Amino acids in red indicate leucine, and isoleucine residues are hallmarks of NES. C. Effect of mutation of leucine 33 and 34 in NES function. (a) Localization of GFP-Tus (1–77) and (b) GFP-Tus (1–77:L33A–L34A double mutants) in PC3 cells. D. Distribution of GFP proteins in PC3 cell line.

While we were mapping the NLS region of Tus, we noticed that deletion of the carboxy terminus (GFP-Tus ΔC, amino acids 218 to 309) caused fluorescence of GFP to concentrate mainly in the cytoplasm (Figure 1). This result suggested the presence of a nuclear export signal (NES) within the first 217 amino acids of Tus. To narrow down the region required for nuclear export, further deletions were made and the distribution of fluorescence was assessed. As can be seen in Figure 2, the NES region is located within the first 77 amino acids of Tus. We examined the primary amino acid sequence of Tus in the first 77 amino acids to determine whether it contains a leucine-rich sequence with hydrophobicity, which is the hallmark criterion established for an NES [13], [20], [24]. A possible consensus nuclear export sequence is LX _1–3_ LX _2–3_ LXL, where L = leucine and X = any amino acid and the last leucine can be replaced by conservative substitutions (isoleucine, valine, etc.). A careful examination of the Tus amino acids sequence showed the presence of several candidate regions. To localize the NES region, several regions of Tus within the first 77 amino acids were cloned at the carboxy terminus of GFP (Figure 4A). The clones containing GFP fused with indicated amino acids portion of Tus were transfected into PC3 cell lines and the distribution of GFP was observed. As indicated in Figure 4A, the NES was localized within 21 amino acids (amino acids 21 and 41 of Tus). Deletion of leucine residue from either end of 21 amino acids abolished the NES function. It appears that all 21 amino acids seem to be required for NES to function. This region contains several clusters of leucine and other hydrophobic amino acids that are hallmarks of NES (Figure 4B). Finally, we have shown that mutation of amino acids L33A and L34A almost completely abolished the function of NES, suggesting the important roles of leucine in NES function (Figure 4C). However, the length of Tus NES is slightly longer than other known NES sequences.

Similar to nuclear import, nuclear export is also thought to use the nuclear pore complexes for exporting large proteins [25], [26], [27], [28]. However, compared to nuclear import, nuclear export is poorly understood. Some proteins that need to shuttle between the nucleus and cytoplasm through the nuclear pore contain both NLS and NES [27], [28]. Examples include the Rev protein of HIV1 and the nuclear factor of activated T cells (NFAT). The Rev protein plays a key role in the regulation of viral expression. NFAT is the target of immunosuppressive drugs widely used in organ transplantation. However, the presence of both NLS and NES in a bacterial protein is unexpected and very unusual, as there is no nuclear membrane for protein translocation between nucleus and cytoplasm.

To understand the mechanism of nuclear export of Tus, we examined the effect of leptomycin, an inhibitor of CRM-1 [13], [14] mediated nuclear export. Our result indicates that Tus NES is probably exported through other mechanism unknown to us at this time. It is possible the Tus uses the same mechanism used by two other known proteins, Tax protein of HTLV-1 [29] and Smad3 [30]. Both proteins contain similar leucine-rich NES for nuclear export as Tus.

### Protein Delivery by Tus and Tus-NLS Functions Similar to TAT

**Figure 5:**
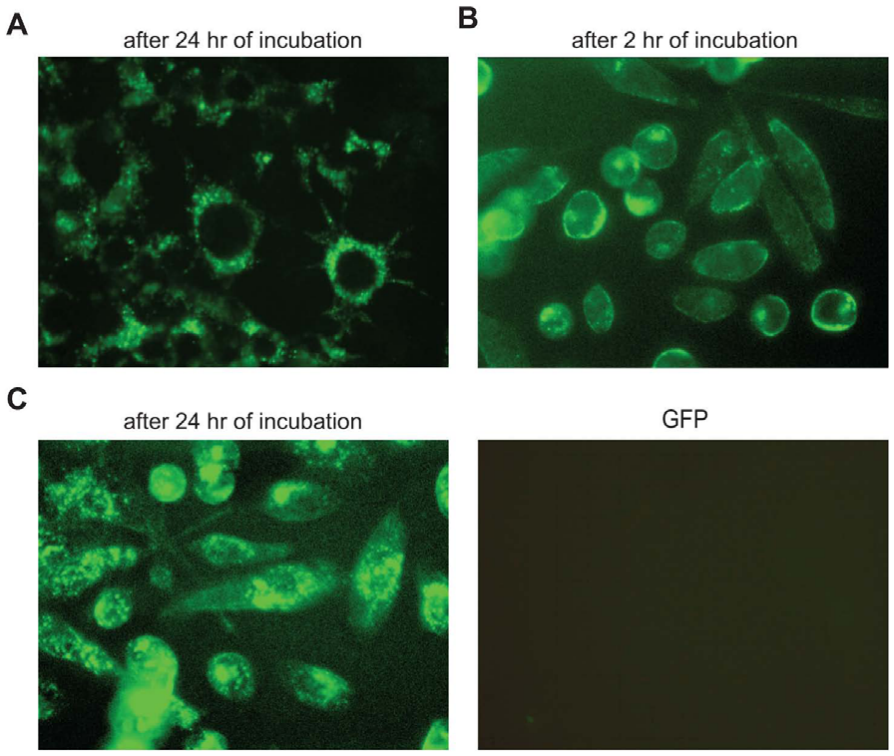
Protein delivery using GFP-Tus and GFP-Tus NLS fusion protein. A. Internalization of GFP-Tus fusion protein in PC3 cell line after 24 hrs post-addition of the fusion protein. B. Internalization of GFP-Tus NLS fusion protein after 2 hrs; or C. 24 hrs post-addition of the fusion protein. D. Addition of GFP fusion protein (control) showed no internalization (negative control).

The direct introduction of proteins into cells may be useful to study cell cycle regulation, control of apoptosis, and transcription regulation. In addition, protein transduction has been shown to be an effective and safer way of delivering biologically active proteins into cells to correct diseases. The plasma membrane is the natural barrier that excludes proteins among other molecules and seems to be the limiting factor in the development of effective protein delivery systems. Over the years, several proteins, including HIV-1 TAT, HSV1-VP22, and Drosophila-Antp, and peptides derived from those proteins have been shown to have the capability to travel (transduce) through plasma membranes [17], [31], [32]. However, most of the internalized proteins end up in endosomal vesicles, and only a small portion were shown to be released. All of these proteins and peptides were derived from eukaryotic sources. To date, there has been no example of any bacterial protein(s) possessing similar attributes. Cre, though it contains a putative NLS sequence, has been shown to transduce very poorly unless it was fused to TAT and another NLS sequence [33]. To our surprise, we have repeatedly found that when purified GFP-Tus fusion protein (∼62 kDa) was included in the culture media, the fusion protein was internalized very rapidly into the PC3 (prostate cancer) human cell line (Figure 5A). Similarly, fusion protein containing GFP plus a 9-amino acid NLS sequence of Tus has similar activity and helps transduce the GFP passenger protein from the culture media to inside of PC3 cells in a time-dependent manner (Figure 5B and C). In contrast, no internalization (GFP fluorescence) was observed when equal amount of purified GFP (without NLS) was used under identical condition (Figure 5D).

We have demonstrated the presence of both nuclear targeting (NLS) and nuclear exporting (NES) signals within a single bacterial protein, Tus. This perhaps is the first known bacterial protein to contain both signals normally present in some mammalian proteins required for shuttling between nucleus and cytoplasm. Both NLS and NES contain putative consensus-like sequences. Why would a bacterial protein like Tus contain determinants that efficiently direct it towards the nucleus or the cytoplasm? Since Tus is a DNA binding protein, initially it was thought to be fortuitous and perhaps due to simple diffusion. However, our results suggest that Tus indeed contains a putative NLS signal and that mutations of essential amino acids in this sequence completely abolished nuclear targeting. NLS sequences often overlap with DNA binding regions. Although the putative NLS region identified in Tus contains some DNA (*Ter*) binding amino acids, we have shown that DNA binding is not essential for nuclear targeting. However, the biological significance of the presence of both NLS and NLS in Tus is not known and any suggestions would be speculative.

It is also noteworthy that full-length Tus fused to GFP directs mostly in the nucleus, suggesting that the NLS is a dominant signal, compared to NES. In a blast search (NCBI GCG-Lite plus parallel Fasta search for protein sequence), we have found that the 21-amino acids NES of Tus has a sequence identity of about 83% with both human and mouse transcription factor (E2F6) and about 75% identity with human and mouse nuclear factor of kappa light polypeptide gene enhancer in B-cells 2 (NFKb2) and Drosophila DNA polymerase subunit α B. In addition, it has various levels of identity with many more human and other mammalian proteins. We do not know if there is any relevance to this similarity. In addition, at this time, we do not know of any biological reasons or significance for the presence of these unusual properties in Tus. We are currently investigating if there is any biological relevance of this discovery.

In addition to finding NLS and NES in Tus protein, our experiments suggest that full-length Tus and its 9-residue NLS peptides may be useful for protein delivery into mammalian cells. GFP fused to either full-length or its NLS is capable of internalization in PC3 or 293 cells (ATCC) within 2 hours after the addition of fusion proteins. We believe that this is the first example of a bacterial protein-mediated protein delivery into mammalian cells and may be added to the list of known mammalian proteins with similar attributes. At this time, we do not know of any biological significance of Tus having NLS or NES. It is possible that the presence is completely fortuitous. Currently, extensive study is being done to elucidate the mechanism of protein transport and release of passenger protein from the endosomal vesicle.
